# Treatment patterns, outcomes and healthcare resource utilization of obstructive hypertrophic cardiomyopathy in England

**DOI:** 10.1002/ehf2.15213

**Published:** 2025-02-12

**Authors:** Faizel Osman, Carla L. Zema, Michael Hurst, Belinda Sandler, Florence Brellier, Ovie Utuama, Oksana Kirichek, John Houghton, Teresa Lemmer, Maite Tome Esteban

**Affiliations:** ^1^ Institute of Cardio‐Metabolic Medicine University Hospitals Coventry and Warwickshire NHS Trust Coventry UK; ^2^ Warwick Medical School University of Warwick Coventry UK; ^3^ Bristol Myers Squibb Princeton New Jersey USA; ^4^ Bristol Myers Squibb Uxbridge UK; ^5^ Health Economics and Outcomes Research Ltd Cardiff UK; ^6^ Cardiovascular Clinical Academic Group, St George's Hospital NHS Foundation Trust London UK; ^7^ St George's University of London London UK

**Keywords:** Clinical outcomes, Healthcare resource utilization, Hypertrophic cardiomyopathy, Obstructive hypertrophic cardiomyopathy, Patient characteristics, Treatment patterns

## Abstract

**Aims:**

Describe patient characteristics, treatment patterns, clinical outcomes, healthcare resource utilization (HCRU) and medical costs associated with patients who were diagnosed with obstructive hypertrophic cardiomyopathy (HCM) in clinical practice in England.

**Methods and results:**

This observational, retrospective, cohort study of adults who were diagnosed with obstructive HCM in routine clinical practice in England used electronic health records from Clinical Practice Research Datalink (CPRD) GOLD/Aurum and linked Hospital Episode Statistics (HES) databases (1 April 2007 to 30 October 2020). Adults (≥18 years at index date) with at least one diagnosis code (ICD‐10, Read, SNOMED, or OPCS) indicative of HCM with ≥1 year of continuous registration in CPRD, data of acceptable research quality and eligibility for HES linkage were included. Outcomes from the obstructive HCM cohort were stratified by New York Heart Association (NYHA) class at baseline and during follow‐up. Owing to the paucity of NYHA coding, patients with obstructive HCM and no record of NYHA class were assigned a proxy NYHA classification using an algorithm that considered patient symptoms and treatments. The study included 6440 patients in the overall HCM cohort with a mean follow‐up duration of 4.84 [standard deviation (SD): 2.95] years. The study population was predominantly male (61.9%) and white (79.1%), with a mean (SD) age of 61.02 (15.61) years. The proportion of patients with obstructive HCM who had a pre‐specified prior medical condition relevant to understanding disease burden increased with higher NYHA class (66.5% vs. 83.0% for NYHA class I and NYHA class II+, respectively), as did the proportion of patients with at least one baseline active prescription for cardiovascular‐related medication. Among patients with at least one record of a prescription for the treatment of symptomatic obstructive HCM, 41.7% experienced a treatment change during the follow‐up period. Atrial fibrillation or flutter, ischaemic stroke and heart failure were the most observed clinical events among patients in the obstructive HCM cohort, and the first in‐study incidence of these events increased with higher NYHA class. Total HCRU costs per patient‐year increased from £3033 to £4517 for NYHA classes I and II+, respectively, with secondary care costs consistently being the main driver in the obstructive HCM cohort.

**Conclusions:**

Obstructive HCM is associated with a large clinical and economic burden in England, and this burden increases with higher NYHA class. These findings support the need for new and more effective strategies for the management of HCM.

## 1 Introduction

Hypertrophic cardiomyopathy (HCM) is a chronic, often progressive disease. About half of all HCM cases are caused by an inherited genetic mutation,^1^ making HCM the most commonly inherited cardiovascular disorder.^2^ The disease is characterized by left ventricular (LV) hypertrophy that is unexplained by loading conditions^1^ and has a reported prevalence of 1 in 500 people.^3^ HCM can be classified as obstructive or non‐obstructive based on the presence or absence of LV outflow tract obstruction (LVOTO).^4^ LVOTO is associated with exacerbation of disease progression and an increased risk of cardiac complications and death.^5^, ^6^ It has been estimated that obstructive HCM accounts for up to two‐thirds of all diagnosed HCM cases.^7^

The clinical profile of obstructive HCM is heterogeneous. Some patients may be asymptomatic; whereas, others may experience a wide spectrum of symptoms including dyspnoea, angina, fatigue and pre‐syncope or syncope.^8^ The symptoms of HCM can be debilitating and can considerably impair patients' physical activity, mental health, ability to work and general quality of life.^9^ HCM can also lead to a wide range of complications; studies using data from electronic health records (EHRs) have shown that patients with HCM have a higher risk of ventricular arrhythmias, cardiac arrest or sudden cardiac death, heart failure and atrial fibrillation (AF) than the general population.^10^, ^11^, ^12^ Increased severity of physical limitations in patients with obstructive HCM, as assessed by cardiologists using the New York Heart Association (NYHA) functional classification system, is associated with an increased risk of all‐cause mortality, all‐cause hospitalization, cardiovascular‐related hospitalization and incident AF or flutter.^13^ This burden of disease can be associated with high healthcare resource utilization (HCRU) and medical costs, particularly for patients with obstructive HCM.^14^

Clinical practice guidelines for the management of obstructive HCM are largely consensus‐based owing to the historical lack of clinical trials in this disease area because, until recently, there have been no specific pharmacological treatments developed to target the underlying pathophysiology of the disease. Older pharmacological therapies [such as beta‐blockers (BBs), calcium channel blockers (CCBs) and disopyramide] may be used predominantly ‘off‐label’ to provide symptomatic relief, with most recommendations assigned an evidence level of B or C.^1^, ^15^, ^16^ Although non‐pharmacological septal reduction therapies (SRTs) can offer long‐term benefits for patients with severe obstructive HCM, the specialist nature of these procedures means that access can be limited and there is a risk of procedure‐related complications.^15^ Many patients are not eligible for SRTs,^17^ may have difficulty accessing specialty centres or may choose not to undergo invasive procedures.^18^

Despite the significant clinical and economic burden of obstructive HCM demonstrated in the United States,^14^, ^19^ there are considerable evidence gaps regarding the epidemiology, patient characteristics, clinical outcomes, treatment patterns and economic burden of obstructive HCM in real‐world clinical practice in other regions, including England. Furthermore, although prior population‐based cohort data for the period 1997–2010 have been published in England,^10^ more recent data specific to the health and economic burden of obstructive HCM in current clinical practice in England are lacking. Addressing these evidence gaps is important to optimize disease management, particularly given the emergence of novel therapies such as cardiac myosin adenosine triphosphatase (ATPase) inhibitors. The aim of this study is to describe the patient characteristics, treatment patterns, clinical outcomes, HCRU and medical costs associated with patients diagnosed with obstructive HCM in current clinical practice in England.

## 2 Methods

### 2.1 Study objectives

The primary objective was to describe patient characteristics at HCM diagnosis with stratification by HCM subtype (obstructive, non‐obstructive or other/unspecified). The secondary objectives were to assess the treatment patterns and pathways, clinical outcomes and HCRU and associated costs among patients with obstructive HCM, stratified by NYHA class.

### 2.2 Study design and data source

This observational, retrospective, cohort study of patients diagnosed with HCM in routine clinical practice in England, leveraged data collected in EHRs from the Clinical Practice Research Datalink (CPRD) GOLD/Aurum and linked Hospital Episode Statistics (HES) databases between 1 April 2007 and 30 October 2020 (study period). The earliest HCM diagnosis considered was 1 April 2009, allowing a 2‐year look‐back period to collect patient baseline characteristics.^20^, ^21^ The CPRD database contains anonymized patient data from a sample of primary care practices in the United Kingdom that use Vision software and EMIS software to inform the CPRD GOLD and CPRD Aurum primary care databases, respectively. Data from CPRD were linked to HES admitted patient care, HES outpatient, HES accident and emergency and HES diagnostic imaging datasets. HES data are only available for England, so the setting of this study was restricted to England owing to data linkage. Linked mortality data from the Office for National Statistics death registrations were used to assess cause‐specific mortality. All data linkage was performed by CPRD before data access. Given that many general practitioner (GP) practices are moving from the CPRD GOLD database to the CRPD Aurum database, CPRD performed de‐duplication from GOLD. The Independent Scientific Advisory Committee (ISAC) approved the study protocol (ISAC application reference 21_000342) on 4 September 2021.

### 2.3 Patient population

The study included adults with at least one diagnosis code (ICD‐10, Read, SNOMED or OPCS codes) in CPRD or HES indicative of obstructive HCM, non‐obstructive HCM, or other/unspecified HCM during the study period. Other eligibility criteria were age of 18 years or older at index date (day 0), ≥1 year of continuous registration in the CPRD before index date, data of acceptable research quality as determined by CPRD and eligibility for HES linkage. Treatment patterns and clinical outcomes were assessed in patients with HCM with ≥1 year of follow‐up from index date or death if within 1 year of index date. Patients were excluded from the study if they had a record of any of the following conditions during the study period: hypertensive heart disease, aortic stenosis, athlete's heart, storage disease, Takotsubo cardiomyopathy, Anderson–Fabry disease, Pompe's disease or amyloidosis.

The index date was defined as the earliest date during the study period at which patients had a diagnosis code for HCM. Baseline characteristics were collected during a 2‐year pre‐diagnosis period, meaning that the earliest index date for cohort assignment was 1 April 2009 (index period). The follow‐up period for each patient was defined as the time from index date to one of the following (whichever occurred first): the end of the study period (30 October 2020), the date of death (if applicable), the general practice last collection date, or the date that the patient's registration at the practice ended. Some patients (*n* = 22) had diagnosis codes relating to both obstructive HCM and non‐obstructive HCM. For the purpose of this analysis, patients with diagnosis codes for both obstructive HCM and non‐obstructive HCM were assigned to the obstructive HCM cohort, with the index date being the date of the earliest obstructive HCM diagnosis. Patients with a diagnosis code that did not specify obstructive or non‐obstructive HCM (i.e., unspecified) were combined with the non‐obstructive HCM cohort to align with current HCM ICD‐10 codes.

### 2.4 Statistical methods and analysis

#### 2.4.1 Patient characteristics

Patient characteristics were summarized using mean, median and standard deviation (SD) for continuous variables and using frequency counts and proportions for categorical variables, on or before the index date. Time‐dependent variables and event counts closest to, on, or before the index date and within a 2‐year pre‐diagnosis period were used. To describe the general health state of patients with HCM at baseline, a list of pre‐specified prior medical conditions considered relevant to understanding the clinical and economic burden of HCM were evaluated. This list was developed based on expert clinical opinion of the most common comorbid conditions observed in patients with HCM in clinical practice and included: asthma, AF or flutter, cardiac arrest, cardiac dysrhythmias, chronic kidney disease, conduction disorders, chronic obstructive pulmonary disease, previous deep vein thrombosis/pulmonary embolism, depression, dilated cardiomyopathy, heart transplant, ventricular assist device, previous stroke or transient ischaemic attack, SRT, peripheral vascular disease, ischaemic heart disease, myocardial infarction, diabetes, implantable cardioverter‐defibrillator insertion, pacemaker, hypertension, obstructive HCM family history. Baseline active prescriptions for cardiovascular‐related medications, using a 6‐week pre‐diagnosis period, were also assessed.

Although NYHA class is used by cardiologists to assess symptoms and functional limitations, a feasibility analysis run by CPRD found that only 1.26% of patients in the primary care datasets had a NYHA classification documented by associated Read code in their records. To address this limitation, a stepwise decision tree algorithm was developed in collaboration with two practising cardiologists in the United Kingdom with experience in the treatment and management of patients with obstructive HCM. All patients with a recorded NYHA class code were assigned to the corresponding NYHA class I–IV at baseline. For patients without a recorded NYHA class code, patients were assigned to a baseline NYHA class based on their record of prescribed medications from day −2 up to and including day 28 from index. Adjustments were made based on relevant recorded symptoms (breathlessness, fatigue, oedema, palpitations, dizziness, syncope, chest pain, angina, pre‐syncope and tachycardia). A time‐varying algorithm was used to assess changes in NYHA classification over time, applied daily. This followed the stepwise approach used by the baseline algorithm, with some minor differences in the look‐back period and asymptomatic correction (i.e., symptomatic patients [NYHA class II+] could not become asymptomatic [NYHA class I] without intervention).

#### 2.4.2 Treatment patterns and pathways

Treatment patterns and pathways, defined as at least one prescription for the respective therapy or therapeutic class and non‐zero days' supply information, were assessed at the product level and at the therapeutic class level. As the patient cohort did not exclusively include newly diagnosed patients, the term ‘initial treatment’ refers to the first record of prescription following the first HCM diagnosis recorded within the study period. BBs, non‐dihydropyridine CCBs and disopyramide were considered obstructive HCM‐specific treatments.^15^, ^16^ Cumulative risk plots described time to any treatment change (augmentation, discontinuation, no treatment), time to treatment augmentation (addition of a different class of medication to an existing prescription), time to treatment discontinuation, time to no treatment, and the number and frequency of treatments. The duration of time each patient received a given line of treatment was summarized using means, medians and SDs.

#### 2.4.3 Clinical outcomes

Clinical outcomes were evaluated as events per patient‐year and the time from the index date to the first occurrence of an event. The following events were only considered at first occurrence as they are chronic conditions/events thought not to be repeatable: AF or flutter, cardiac dysrhythmias, conduction disorders, heart failure, dilated cardiomyopathy, all‐cause mortality, implantable cardioverter‐defibrillator and pacemaker insertion. Events with acute or immediate clinical presentation were considered as repeated events, including myocardial infarction, stroke, ventricular tachycardia, SRT, deep vein thrombosis/pulmonary embolism, and cardiac arrest/ventricular fibrillation, and were considered unique if a code was observed ≥30 days apart. Incidence rates were calculated using unadjusted generalized estimating equation models with a negative binomial distribution and first‐order autoregressive correlation structure and stratified by time‐varying NYHA class. The Kaplan–Meier method was used to assess the time to first occurrence of event.

#### 2.4.4 Healthcare resource utilization and costs

To quantify the economic burden, HCRU per patient‐year during the follow‐up period was calculated and stratified by time‐varying NYHA class. Primary care resources were assessed based on the number of nurse practice consultations, GP consultations, telephone consultations, and out‐of‐hours consultations sourced from the CPRD GOLD/Aurum consultation files in combination with the staff files. Secondary care resources included hospital inpatient admissions, day cases, elective stays, non‐elective stays, outpatient visits, emergency visits and critical care. Tests, diagnoses and procedures identified via OPCS‐4.9 codes in HES procedures or Read codes from CPRD datasets were used to identify events. With the exception of inpatient costs, the costs associated with HCRU were calculated based on total counts of corresponding service activity defined in HES data multiplied by activity type (unit) costs obtained from the National Schedule of National Health Service (NHS) Costs – Year 2019–20 (NHS Trusts and NHS Foundation Trusts) in England.^22^ These costs were inflated according to the Personal Social Services Research Unit report of February 2021.^23^ Inpatient costs were calculated using the HES healthcare resource groups (HRG) table to pull the total cost of the relevant inpatient visits using HRG currency codes, subsequently summarized for elective and non‐elective inpatient admissions and day cases.

## 3 Results

### 3.1 Cohort development

Of the 35 886 patients identified in the CPRD GOLD/Aurum HES linked dataset, 6440 patients (17.95%) met the eligibility criteria for inclusion in the overall HCM cohort. Mean follow‐up duration was 4.84 (SD: 2.95) years. Of these eligible patients, 3730 (57.92%) and 2710 (42.08%) were assigned to the obstructive and non‐obstructive/unspecified HCM cohorts, respectively (*Figure* *S1*). Of note, 22 patients who had a diagnosis of non‐obstructive HCM followed by a diagnosis of obstructive HCM during the follow‐up period were assigned to the obstructive HCM cohort for the purpose of this analysis.

### 3.2 Patient characteristics and New York Heart Association class distribution

At baseline, the overall HCM cohort (*n* = 6440) had a mean age of 61.02 (SD: 15.61) years and the study population were predominantly male (61.9%) and white (79.1%). Baseline demographics were numerically similar across the obstructive HCM cohort (*n* = 3730) and the non‐obstructive HCM cohort (*n* = 2710) (*Table* *S1*). The proportion of patients with at least one prior medical history record was similar between the non‐obstructive (77.5%) and obstructive (78.7%) HCM subtypes (*Table* *S1*). Most patients in both the non‐obstructive (76.7%) and obstructive (81.7%) cohorts had at least one baseline active prescription (*Table* *S2*).

At baseline, the distribution of the obstructive HCM cohort (*n* = 3730) by NYHA class was 966 (25.9%), 1264 (33.9%), 1407 (37.7%) and 93 (2.5%) for NYHA classes I, II, III and IV, respectively. The study population had numerically similar ethnicity and sex distributions regardless of baseline NYHA class. The mean age of patients increased with higher NYHA class, from 57.7 (SD: 16.3) years in NYHA class I to 66.7 (SD: 13.5) years and 63.8 (SD: 13.2) years in NYHA class III and IV, respectively (*Table* *1*). When comparing the mean age of asymptomatic patients (NYHA class I) with symptomatic patients (NYHA class II+: 62.5 [SD: 15.1] years) a similar increase was observed. The proportion of patients with obstructive HCM who had at least one pre‐specified prior medical condition relevant to understanding disease burden increased with higher NYHA class, from 66.5% in class I to 83.0% in NYHA class II+. The prevalence of some conditions increased with higher NYHA classes (*Table* *1*), including AF or flutter (9.2% vs. 20.7%), type 2 diabetes (7.0% vs. 18.5%) and stroke (7.4% vs. 14.2%) when comparing NYHA class I and III, respectively, for example.

**Table 1:** Baseline patient demographics of the obstructive HCM cohort, stratified by NYHA classification at baseline.

Baseline characteristics	All obstructive HCM (*n* = 3730)	NYHA class I (*n* = 966)	NYHA class II (*n* = 1264)	NYHA class III (*n* = 1407)	NYHA class IV (*n* = 93)
Age, years, mean (SD)	61.2 (15.5)	57.7 (16.3)	57.7 (15.4)	66.7 (13.5)	63.8 (13.2)
Male, *n* (%)	2257 (60.5)	637 (65.9)	811 (64.2)	752 (53.5)	57 (61.3)
White, *n* (%)	3013 (80.8)	788 (81.6)	1022 (80.9)	1121 (79.7)	82 (88.2)
Asian/British Asian ethnicity, *n* (%)	333 (8.9)	85 (8.8)	124 (10.0)	115 (8.2)	7 (7.5)
Black/Black British ethnicity, *n* (%)	233 (6.3)	NR^a^	59 (4.7)	118 (8.4)	NR^b^
Mixed ethnicity, *n* (%)	43 (1.2)	NR^a^	22 (1.7)	14 (1.0)	NR^b^
Other ethnicity, *n* (%)	60 (1.6)	10 (1.0)	25 (2.0)	25 (1.8)	0 (0.0)
Age‐adjusted CCI score, mean (SD)	2.96 (2.40)	2.32 (2.19)	2.36 (2.18)	3.85 (2.39)	4.38 (2.65)
Patients with at least one documented medical condition^c^, *n* (%)	2937 (78.7)	642 (66.5)	912 (72.2)	1294 (92.0)	89 (95.7)
Asthma, *n* (%)	481 (12.9)	94 (9.7)	160 (12.7)	207 (14.7)	20 (21.5)
AF or flutter, *n* (%)	573 (15.4)	89 (9.2)	156 (12.3)	291 (20.7)	37 (39.8)
Cardiac arrest, *n* (%)	40 (1.1)	NR^b^	24 (1.9)	NR^a^	0 (0)
Cardiac dysrhythmias^d^, *n* (%)	431 (11.6)	54 (5.6)	180 (14.2)	172 (12.2)	25 (26.9)
CKD, *n* (%)	394 (10.6)	77 (8.0)	65 (5.1)	230 (16.4)	22 (23.7)
Conduction disorders, *n* (%)	317 (8.5)	49 (5.1)	99 (7.8)	151 (10.7)	18 (19.4)
COPD, *n* (%)	287 (7.7)	28 (2.9)	76 (6.0)	156 (11.1)	27 (29.0)
Deep vein thrombosis/pulmonary embolism, *n* (%)	56 (1.5)	NR^a^	17 (1.3)	23 (1.6)	NR^b^
Depression, *n* (%)	327 (8.8)	62 (6.4)	106 (8.4)	145 (10.3)	14 (15.1)
Dilated cardiomyopathy, *n* (%)	60 (1.6)	5 (0.5)	11 (0.9)	32 (2.3)	12 (12.9)
Heart transplantation, *n* (%)	NR^b^	0 (0)	NR^b^	0 (0)	0 (0)
Hypertension, *n* (%)	1778 (47.7)	416 (43.1)	330 (26.1)	978 (69.5)	54 (58.1)
ICD insertion, *n* (%)	106 (2.8)	9 (0.9)	48 (3.8)	43 (3.1)	6 (6.5)
Ischaemic heart disease, *n* (%)	956 (25.6)	146 (15.1)	299 (23.7)	470 (33.4)	41 (44.1)
Myocardial infarction, *n* (%)	296 (7.9)	52 (5.4)	93 (7.4)	138 (9.8)	13 (14.0)
Obstructive HCM family history, *n* (%)	65 (1.7)	33 (3.4)	16 (1.3)	NR^a^	NR^b^
Pacemaker, *n* (%)	146 (3.9)	20 (2.1)	56 (4.4)	64 (4.6)	6 (6.5)
Peripheral vascular disease, *n* (%)	56 (1.5)	NR^a^	10 (0.8)	35 (2.5)	NR^b^
SRT, *n* (%)	6 (0.2)	NR^b^	NR^b^	NR^b^	0 (0)
Stroke, *n* (%)	405 (10.9)	71 (7.4)	111 (8.8)	200 (14.2)	23 (24.7)
T1DM, *n* (%)	29 (0.8)	NR^b^	6 (0.5)	18 (1.3)	NR^b^
T2DM, *n* (%)	460 (12.3)	68 (7.0)	102 (8.1)	260 (18.5)	30 (32.3)
Unspecified diabetes, *n* (%)	33 (0.9)	NR^b^	5 (0.4)	24 (1.7)	NR^b^
Transient ischaemic attack, *n* (%)	58 (1.6)	NR^a^	18 (1.4)	24 (1.7)	NR^b^
Ventricular assist device, *n* (%)	0 (0)	0 (0)	0 (0)	0 (0)	0 (0)
Follow‐up, years, mean (SD)	5.2 (3.0)	5.1 (3.0)	5.5 (3.1)	5.1 (3.0)	4.4 (2.6)

*Note*: Ethnicities will not equate to the sum total owing to missing/unknown data.

Abbreviations: AF, atrial fibrillation; CCI, Charlson's Comorbidity Index; CKD: chronic kidney disease; COPD, chronic obstructive pulmonary disease; HCM, hypertrophic cardiomyopathy; ICD, implantable cardioverter‐defibrillator; ICD‐10, International Classification of Diseases, Tenth Revision; NR, not reported; NYHA, New York Heart Association; SD, standard deviation; SRT, septal reduction therapy; T1DM, type 1 diabetes mellitus; T2DM, type 2 diabetes mellitus.

^a^ Secondary suppression.

^b^ Primary suppression applied to cells with < 5 observations.

^c^ Documented medical conditions that occurred in the 2 years before index obstructive HCM diagnosis, based on a pre‐specified list informed by expert clinical opinion of the most common comorbid conditions observed in clinics considered relevant to understand the clinical and economic burden of HCM: asthma, AF or flutter, cardiac arrest, cardiac dysrhythmias, CKD, conduction disorders, COPD, previous deep vein thrombosis/pulmonary embolism, depression, dilated cardiomyopathy, heart transplant, ventricular assist device, previous stroke or transient ischaemic attack, SRT, peripheral vascular disease, ischaemic heart disease, myocardial infarction, T1DM, T2DM, ICD insertion, pacemaker, hypertension, obstructive HCM family history. Patients may have had comorbid medical conditions; therefore, the listed medical conditions do not equate to the sum total of ‘Patients with at least one documented medical condition’.

^d^ Includes paroxysmal tachycardia (ICD‐10: I47), atrial fibrillation and flutter (ICD‐10: I48), and other cardiac arrhythmias (ICD‐10: I49).

The proportion of patients with at least one baseline active prescription was 58.5% for patients in NYHA class I and 81.3% in class II (class III/IV percentages were suppressed as per CPRD guidance) (*Table* *2*). Of the cardiovascular‐related medications considered, the most prescribed treatments included blood‐pressure lowering medications, statins and antiplatelet medication.

**Table 2:** Baseline active prescriptions for cardiovascular‐related medication in the obstructive HCM cohort, stratified by NYHA class at baseline.

Medication, *n* (%)	All obstructive HCM (*n* = 3730)	NYHA class I (*n* = 966)	NYHA class II (*n* = 1264)	NYHA class III (*n* = 1407)	NYHA class IV (*n* = 93)
Patients with at least one known baseline active prescription	3048 (81.7)	565 (58.5)	1027 (81.3)	NR^a^	NR^a^
Blood‐pressure lowering	2866 (76.8)	NR^a^	972 (76.9)	1336 (95.0)	NR^a^
Amiodarone	193 (5.2)	28 (2.9)	57 (4.5)	92 (6.5)	16 (17.2)
Statins	1760 (47.2)	325 (33.6)	489 (38.7)	882 (62.7)	64 (68.8)
Antiplatelet	1291 (34.6)	221 (22.9)	373 (29.5)	657 (46.7)	40 (43.0)
Anticoagulant	599 (16.1)	87 (9.0)	136 (10.8)	324 (23.0)	52 (55.9)
Aspirin	1194 (32.0)	206 (21.3)	346 (27.4)	606 (43.1)	36 (38.7)

*Note*: Baseline active prescriptions were based on prescribing dates before the index date within a 6‐week look‐back period. The look‐back period for all other characteristics and outcomes is 2 years before the index date.

Abbreviations: HCM, hypertrophic cardiomyopathy; NR, not reported; NYHA, New York Heart Association.

^a^ Secondary suppression.

### 3.3 Treatment patterns and pathways

For patients with obstructive HCM, 82.7% had at least one recorded prescription for the treatment of symptomatic obstructive HCM (i.e., a BB, a CCB or disopyramide). The number of prescriptions for the treatment of obstructive HCM per patient per calendar year was consistent from the index date to the end of the follow‐up period, with means of 1.2 and 1.1 different therapy classes prescribed in 2010 and 2020, respectively. Of those patients with at least one recorded prescription associated with the treatment of symptomatic obstructive HCM (*n* = 3084), the most common initial treatments observed by therapeutic class were BB and CCB monotherapies, which were prescribed to 79.6% and 12.9% of patients, respectively (*Table* *3*). Conversely, the least common initial monotherapy treatment observed was disopyramide (0.6%). Of the initial treatments observed, dual therapy with different therapeutic classes was prescribed to 5.4% of patients with a recorded prescription for the treatment of symptomatic obstructive HCM. Further detail on the most common initial treatments observed at product level is available in *Table* *S3*.

**Table 3:** Initial treatment observed and summary of first treatment changes by therapeutic class in the obstructive HCM cohort.

	Patients, *n* (%)
Initial treatment observed^a^	
Patients with any HCM‐specific treatment	3084 (82.7)
BB monotherapy	2456 (79.6)
CCB monotherapy	399 (12.9)
BB + CCB dual therapy	83 (2.7)
BB + disopyramide dual therapy	66 (2.1)
BB + BB^b^ dual therapy	32 (1.0)
CCB + disopyramide dual therapy	19 (0.6)
Disopyramide monotherapy	18 (0.6)
BB + CCB + disopyramide triple therapy	5 (0.2)
CCB + CCB dual therapy^b^	NR^c^ (NR^c^)
BB + BB^b^ + disopyramide triple therapy	NR^c^ (NR^c^)
BB + BB^b^ + CCB triple therapy	NR^c^ (NR^c^)
First treatment change	
No treatment (≥90‐day gap)	557 (18.1)
Augment: monotherapy to combination (e.g., BB to BB + CCB) or add to existing combination (e.g., BB + BB to BB + BB + CCB)	296 (9.6)
Discontinuation: combination to monotherapy (e.g., BB + CCB to BB) or drop one from a combination (e.g., BB + BB + CCB to BB + CCB)	146 (4.7)
Switch: between drug classes (e.g., BB to CCB)	140 (4.5)
Switch: change within drug class (e.g., BB to BB)	142 (4.6)
Switch: change with an addition (e.g., BB to different BB + CCB)	5 (0.2)

*Note*: In total, 646 patients in the obstructive HCM cohort had no record of HCM‐specific treatment (BB or CCB or disopyramide).

Abbreviations: BB, beta‐blocker; CCB, calcium channel blocker; HCM, hypertrophic cardiomyopathy; NR, not reported.

^a^ First treatment recorded during study period.

^b^ Two different drugs within the same therapeutic class.

^c^ Primary suppression applied to cells with <5 observations.

Of those with at least one record of a prescription for the treatment of symptomatic obstructive HCM, 41.7% of patients experienced a first treatment change. Periods of no treatment (≥90 days) (18.1%) or treatment augmentation (9.6%) were the most common treatment changes observed (*Table* *3*).

**Figure 1:**
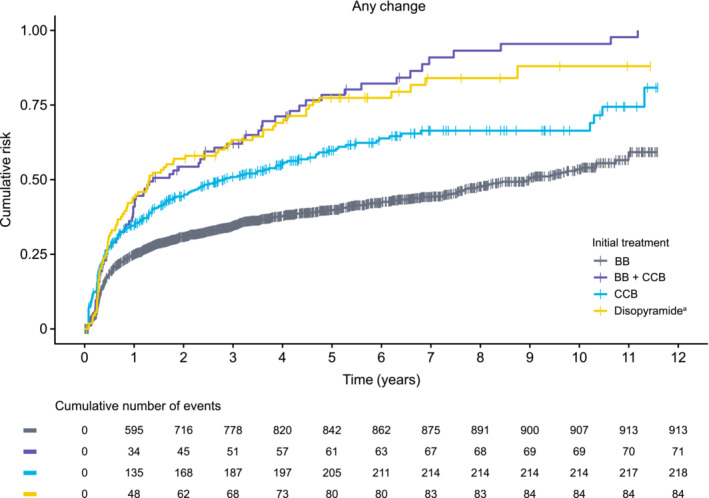
Cumulative risk of any treatment change in the obstructive hypertrophic cardiomyopathy cohort by the initial treatment observed. BB, beta‐blocker; CCB, calcium channel blocker. ^a^Includes disopyramide as monotherapy and in combination therapy.

The cumulative risk of first treatment change (augmentation, discontinuation, or no treatment) in the obstructive HCM cohort was assessed at the therapeutic class level (*Figure* *1*). The proportion of patients with any treatment change over the duration of follow‐up was lowest for those initiated with BB treatment [BB monotherapy and BB + BB; 913/2488 (36.7%)] and highest for those initiated with a combination of BB + CCB therapy (84.5%). It is noteworthy that, within the first year of the observed initial treatments, the greatest proportion of patients with any treatment change was seen in those initiated with disopyramide (monotherapy and in combination therapy; 43.6%) compared with those initiated with BB + CCB (40.5%), CCB (33.6%) or BB (23.9%). Data for cumulative risk of augmentation, discontinuation and no treatment in the obstructive HCM cohort are presented in *Figure* *S2*.

### 3.4 Clinical outcomes

**Figure 2:**
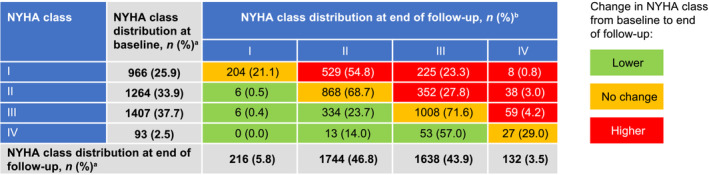
Summary of New York Heart Association (NYHA) class distribution at baseline and end of follow‐up. HCM, hypertrophic cardiomyopathy. ^a^Proportion calculated using obstructive HCM cohort (*n* = 3730). ^b^Proportion calculated using NYHA class distribution at baseline.

A greater proportion of patients with obstructive HCM experienced symptoms of various severity (NYHA class II+) at the end of the follow‐up than at baseline (94.2% vs. 74.1%) (*Figure* *2*). At the end of follow‐up, only 5.8% of patients with obstructive HCM remained asymptomatic (NYHA class I) compared with 25.9% at the time of diagnosis, implying progression of disease. The time‐varying NYHA algorithm showed that patients with obstructive HCM spent the majority of follow‐up time in NYHA classes II or III, with a total of 8558.2 (44.3%) and 8289.3 (42.9%) patient‐years, respectively. This trend was observed at the end of the per‐patient follow‐up, in which patients were predominantly distributed between NYHA classes II and III, with a total of 1744 (46.8%) and 1638 (43.9%) patients, respectively (*Figure* *2*).

**Figure 3:**
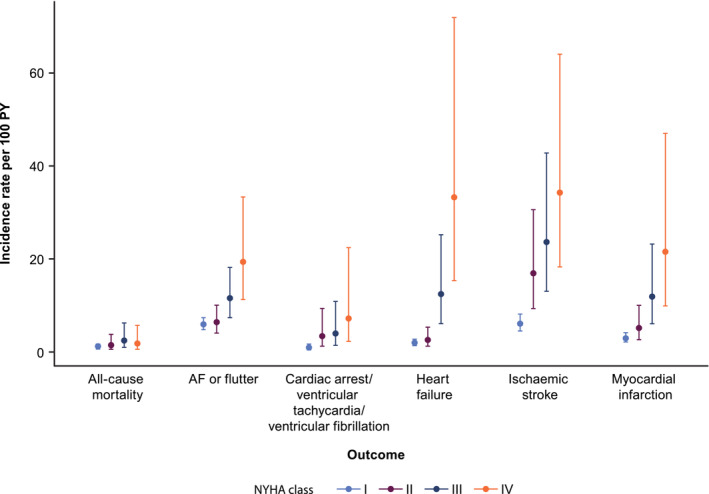
Incidence and 95% confidence interval of events per 100 patient‐years (PY) in the obstructive hypertrophic cardiomyopathy cohort, stratified by time‐varying New York Heart Association (NYHA) class. AF, atrial fibrillation.

The incidence rates of clinical events observed in the obstructive HCM cohort over time, stratified by NYHA class, are presented in *Table* *S4*. AF or flutter, ischaemic stroke and heart failure were the most commonly observed events. Overall, there was a tendency of higher incidence rates for clinical events in higher NYHA classes (*Figure* *3*). The clinical events with strongest difference in incidence rate per 100 patient‐years between NYHA classes I and IV were ischaemic stroke [6.10 (95% confidence interval [CI]: 4.55–8.18) vs. 34.27 (95% CI: 18.34–64.05)], heart failure [1.96 (95% CI: 1.38–2.78) vs. 33.27 (95% CI: 15.39–71.91)], myocardial infarction [3.00 (95% CI: 2.17–4.14) vs. 21.59 (95% CI: 9.91–47.00)], AF or flutter [5.96 (95% CI: 4.80–7.41) vs. 19.41 (95% CI: 11.3–33.35)], and cardiac arrest or ventricular fibrillation or ventricular tachycardia [1.07 (95% CI: 0.65–1.76) vs. 7.19 (95% CI: 2.31–22.44)]. Symptomatic patients (NYHA class II+) consistently had higher incidence rates of clinical events compared with asymptomatic patients (NYHA class I). Lastly, the incidence rate per 100 patient‐years increased for all‐cause mortality from 1.13 (95% CI: 0.72–1.78) for NYHA class I to 2.47 (95% CI: 0.98–6.26) for NYHA class III, but it reduced to 1.81 (95% CI: 0.58–5.72) for NYHA class IV.

### 3.5 Economic burden

For the overall obstructive HCM cohort, total HCRU costs per patient‐year were £4386 (*Table* *4* *)*. Total HCRU costs per patient‐year were £3033 for patients in NYHA class I compared with £7881 for patients in NYHA class IV (*Table* *4*). When comparing asymptomatic vs. symptomatic patients (NYHA class I vs. II+), a similar increase in total HCRU costs was observed (£3033 vs. £4517 per patient‐year, respectively). Secondary care costs consistently accounted for the majority of the total HCRU costs across the obstructive HCM cohort and increased with higher NYHA class (£2355 per patient‐year for NYHA class I vs. £3284 per patient‐year for NYHA class II+). For patients in NYHA class IV, secondary care HCRU was driven primarily by inpatient admissions and outpatient visits, accounting for £3980 and £1506 per patient‐year, respectively (*Table* *S5*). Similarly, total primary care HCRU costs per patient‐year were greater for symptomatic patients (NYHA class II+) compared with asymptomatic patients (NYHA class I) (£398 vs. £209, respectively).

**Table 4:** Total healthcare resource costs for patients with obstructive HCM, stratified by time‐varying NYHA class.

HCRU	All obstructive HCM (*n* = 3730/PY = 19 352		NYHA class I (*n* = 1039/PY = 1709)		NYHA class II (*n* = 2585/PY = 8565)		NYHA class III (*n* = 2418/PY = 8296)		NYHA class IV (*n* = 404/PY = 782)	
	Total cost, £	Cost per PY, £	Total cost, £	Cost per PY, £	Total cost, £	Cost per PY, £	Total cost, £	Cost per PY, £	Total cost, £	Cost per PY, £
Primary care^a^	7 382 460	381	357 910	209	2 682 231	313	3 834 572	462	507 747	649
Secondary care	61 972 989	3202	4 025 136	2355	21 968 729	2565	31 446 374	3791	4 532 749	5796
Tests and procedures	15 524 499	802	800 040	468	5 922 893	692	7 679 343	926	1 122 223	1435
**Total**	84 879 948	4386	5 183 086	3033	30 573 853	3570	42 960 290	5178	6 162 719	7881

Abbreviations: HCM, hypertrophic cardiomyopathy; HCRU, healthcare resource utilization; NYHA, New York Heart Association; PY, patient‐year.

^a^ Out‐of‐hours practice visits are not included.

## 4 Discussion

To address the lack of data describing the epidemiology, disease management, and clinical and economic burden of HCM in England, this retrospective study aimed to evaluate the patient characteristics, treatment patterns, clinical outcomes, HCRU and medical costs associated with patients diagnosed with HCM and its subtypes in routine clinical practice in England using EHRs from CPRD GOLD/Aurum and linked HES databases.

The need for a greater understanding of the current health and economic burden of obstructive HCM in England to inform treatment decision‐making in this population is important, particularly given the upcoming shift in the treatment paradigm. Current standard of care has been in place since the 1980s^24^, ^25^, ^26^; however, this has changed recently with the introduction of cardiac myosin ATPase inhibitors targeting the underlying pathophysiology of obstructive HCM.^1^ Cardiac myosin ATPase inhibitors have demonstrated efficacy in phase 3, randomized controlled trials^27^, ^28^ and have recently been approved by the US Food and Drug Administration and the European Medicines Agency.^29^, ^30^ The 2023 update to the European Society of Cardiology's cardiomyopathy guidelines reflects this evidence, making class IIa, level A recommendations regarding the use of the first‐in‐class cardiac myosin ATPase inhibitor, mavacamten, in patients with symptomatic obstructive HCM.^1^

The clinical profile of HCM is highly variable; many patients can be asymptomatic or have subtle symptoms similar to other conditions such as asthma, anxiety, or mitral valve prolapse, which may lead to misdiagnosis.^31^ A greater understanding of the baseline demographics and characteristics of patients with HCM in clinical practice may increase the awareness of HCM and potentially improve differential diagnosis. The overall HCM cohort was predominantly male (61.9%) and white (79.1%), with a mean age of 61.02 (SD: 15.61) years, which is in line with that described in a previous analysis of linked primary care, hospital and mortality records in patients with HCM in England between 1997 and 2010 [59.0% male, 91.3% white, mean age of 55.8 (SD: 19.9) years].^10^ Within the overall HCM cohort, a greater proportion of patients was defined as having obstructive HCM than non‐obstructive or unspecified HCM (3730 patients vs. 2710 patients), which is similar to a previously reported analysis.^32^ Generally, a higher NYHA class in patients with obstructive HCM was associated with a greater proportion of patients having a prior medical history as well as a greater HCRU including usage of baseline medications. This indicates that patients with a higher NYHA class are more likely to be in poorer health state at baseline than those in a lower NYHA class and are more likely to have higher ongoing HCRU.

For patients with at least one record of a prescription for the treatment of symptomatic obstructive HCM, BB monotherapies were the most frequent initial treatment observed followed by CCB monotherapies, whereas disopyramide was the least commonly prescribed monotherapy. These findings are comparable to those described by a retrospective study based on healthcare claims data from the IBM MarketScan Commercial and Medicare Supplemental database (2009 to 2019), demonstrating that BB monotherapy (70.2%) and disopyramide (2.4%) were the most and least commonly prescribed index date treatment, respectively.^33^ A retrospective database study of the Japan Medical Data Vision database (2016 to 2020) similarly demonstrated that BBs were the most commonly prescribed treatment for obstructive HCM (64.0%), whereas CCBs were prescribed to 25.4% of patients.^34^ The low use of disopyramide observed in this study may be expected given the discrepancies in the reimbursement of disopyramide globally and the known supply issues of disopyramide,^35^ alongside the fact clinical guidelines do not recommend disopyramide as a first‐line treatment and suggest it should be used in combination with BBs or calcium antagonists.^1^

Of the patients with at least one record of a prescription for the treatment of symptomatic obstructive HCM, 41.7% experienced a treatment change during the follow‐up period, suggesting that a substantial proportion of patients receiving current pharmacological treatments require alternative or additional therapies to manage their symptoms. Within the first year post index date, the proportion of patients with a treatment change was greatest for those initiated with disopyramide as monotherapy or in combination therapy, a finding that aligns with a retrospective study reporting that 56.3% of patients initiated with disopyramide had a treatment change within 12 months.^33^ Treatment augmentation was one of the most frequent treatment changes observed among the obstructive HCM cohort, further demonstrating the unmet need for more effective pharmacological therapies, a finding also supported by published literature.^33^ Future research into the cause behind these treatment changes is needed to better understand how current pharmacotherapies fail to manage obstructive HCM effectively. The 2020 American Heart Association/American College of Cardiology guidelines highlighted that ‘there are no known preventive or disease modifying therapies for HCM’ and noted that the use of BBs and CCBs in HCM is largely empiric,^16^ although a recent, small, placebo‐controlled crossover trial of metoprolol in obstructive HCM demonstrated benefit on LVOTO and symptoms.^36^

There is a paucity of data describing the clinical burden of HCM according to disease severity. Using a novel algorithm in this study to assign patients to a proxy NYHA class, the incidence of clinical events was shown to increase with higher NYHA class. This finding aligns with a prior US‐based cohort analysis, which similarly demonstrated that a worse time‐varying NYHA class was associated with a significantly increased risk of all‐cause mortality, all‐cause hospitalization, cardiovascular‐related hospitalization and incident AF or flutter.^37^ These results indicate that the clinical burden of obstructive HCM considerably increases with higher NYHA and, therefore, with disease progression, showing the importance of both diagnosis early in the disease process, and initiation of effective disease management at diagnosis. This is particularly important given that higher NYHA class has been linked to reduced health‐related quality of life.^38^

Alongside the clinical impacts of HCM, the results indicate that obstructive HCM management is associated with significant economic burden, where total costs increased with higher NYHA class. Existing economic analyses of HCM are largely based on US claims data^33^, ^39^, ^40^; this adds a challenge when comparing study results because of the differences in healthcare systems, patient management and baseline demographics. However, findings generally align with prior studies showing that HCRU and costs remain high, particularly in patients with more severe disease.^33^, ^39^, ^40^ Notably, the results here also align with an expert elicitation exercise from cardiologists in the United Kingdom that, despite using a different methodology, arrived at a similar conclusion and adds a level of validity to these findings.^14^ This current analysis reports an average cost (irrespective of NYHA class) of £4386 for the management of obstructive HCM per patient‐year, which is comparable with HCRU costs of other prevalent cardiovascular pathologies in the United Kingdom, such as coronary heart disease (£5530 per patient^41^) and atrial fibrillation (£3731 per patient^42^), though comparisons between such pathologies should be treated with caution due to the different methodologies employed by the studies in question. While this may provide insight into the economic burden of HCM within the cardiovascular landscape, it is important to recognize that HCRU differs between pathologies depending on specific disease characteristics and duration.

There are several limitations to acknowledge for this study. First, owing to the lack of NYHA class coding, the development of an algorithm to assign a proxy NYHA class was required. Although developed using best‐available data, there is the potential for misclassification using this algorithm. Additionally, it is important to note that patients with unspecified HCM (i.e., a diagnosis code that did not specify obstructive or non‐obstructive HCM) were combined with the non‐obstructive HCM cohort for the purpose of this study, aligning with prior analyses which define HCM using ICD‐10 codes *I42.1 obstructive HCM* and *I42.2 nonobstructive or unspecified HCM*.^43^ As a result, only patients with obstructive HCM explicitly coded were included in analyses of the obstructive HCM cohort. There are general methodological limitations inherent to retrospective cohort analyses using data originally collected as part of routine clinical practice, such as inconsistencies in coding, data entry and missing data. In particular, abstracting patients with HCM from clinical records leaves out asymptomatic or patients with mild disease which likely leads to under‐ascertainment of the patient cohort, while missing data may lead to under‐estimation of outcome rates; both will lead to under‐estimation of the true burden of disease. Furthermore, diagnosis codes for dilated cardiomyopathy were observed in patients with HCM. Given these are distinct cardiomyopathies, it is possible that patients with HCM progressed into end‐stage disease, leading to symptoms similar to that of dilated cardiomyopathy and possibly misdiagnosis, signifying a potential limitation of coding practices. Retrospective analyses can only be associative and are unable to assign causality. For this analysis, linked CPRD and HES data were deemed the most robust sources to inform primary care and secondary care coverage in England. However, only patients within the CPRD dataset who were registered with general practices that had agreed to the linkage scheme were eligible for linkage between datasets. The simultaneous use of CPRD GOLD and Aurum datasets may have affected the recording of healthcare activity given the different structure of the primary care consultation records used in both datasets. Lastly, given that the data used for this analysis was restricted to clinical practices in England, these findings may not be generalizable beyond this setting.

This analysis of real‐world data indicates that there is a need for better management options for obstructive HCM, which is a disease associated with a large clinical and economic burden in England. These results contribute to the understanding of the current burden of illness, in which initiating innovative interventions for patients with obstructive HCM may improve clinical outcomes and may have the potential to reduce the HCRU associated with disease management.

## Conflict of interest

Faizel Osman receives research grants from Abbott Medical, Boston Scientific, Bristol Myers Squibb, Creavo Medical Technologies, Medtronic, and British Heart Foundation. Carla L. Zema was a contractor for Bristol Myers Squibb at the time of the study. Michael Hurst, Belinda Sandler, Florence Brellier, and Teresa Lemmer are employees of Bristol Myers Squibb and may own Bristol Myers Squibb stocks or stock options. Michael Hurst and Belinda Sandler attended meetings as employees of Bristol Myers Squibb in relation to this study. Ovie Utuama has a fellowship from Bristol Myers Squibb and attended meetings in relation to this study. Oksana Kirichek and John Houghton are employees of Health Economics and Outcomes Research Ltd which received fees from Bristol Myers Squibb in relation to this study. Maite Tome Esteban has participated in advisory boards for and received consulting fees from Bristol Myers Squibb and Cytokinetics.

## Funding

This work was supported by Bristol Myers Squibb.

## Supporting information


**Table S1.** Baseline patient demographics of the overall HCM cohort stratified by HCM subtype
**Table S2.** Baseline active prescriptions for medication in the overall HCM cohort stratified by HCM cohort
**Table S3.** Top 20 initial treatments observed in the obstructive HCM cohort
**Table S4.** Incidence rates of events per 100 PY in the obstructive HCM cohort stratified by NYHA class
**Table S5.** Healthcare resource use for patients with obstructive HCM, stratified by NYHA class
**Figure S1.** Summary of patient counts through each attrition criterion and HCM cohort.
**Figure S2.** Cumulative risk of treatment augmentation (A), treatment discontinuation (B), and no treatment (C) in the obstructive HCM cohort by initial treatment observed.
