# Supplementary material for: Treatment patterns, outcomes and healthcare resource utilization of obstructive hypertrophic cardiomyopathy in England
Source: ESC Heart Fail. 2025 Feb 12;12(3):2034–46. doi: 10.1002/ehf2.15213 (PMC12055402; doi:10.1002/ehf2.15213)

**Supporting Information**

**Treatment patterns, outcomes, and healthcare resource utilization of obstructive hypertrophic cardiomyopathy in England**

Faizel Osman^1,2^*, Carla L. Zema^3^, Michael Hurst^4^, Belinda Sandler^4^, Florence Brellier^4^, Ovie Utuama^3^, Oksana Kirichek^5^, John Houghton^5^, Teresa Lemmer^4^ and Maite Tome Esteban^6,7^

^1^Institute of Cardio-Metabolic Medicine, University Hospitals Coventry and Warwickshire NHS Trust, Coventry, UK; ^2^Warwick Medical School, University of Warwick, Coventry, UK; ^3^Bristol Myers Squibb, Princeton, NJ, USA; ^4^Bristol Myers Squibb, Uxbridge, UK; ^5^Health Economics and Outcomes Research Ltd, Cardiff, UK; and ^6^Cardiovascular Clinical Academic Group, St George’s Hospital NHS Foundation Trust, London, UK; and ^7^St George’s, University of London, London, UK

*Correspondence to: Faizel Osman. Email: [faizel.osman@uhcw.nhs.uk](mailto:faizel.osman@uhcw.nhs.uk). Telephone: 02476 96 5668. Fax: 02476 96 5657.

**Supplementary tables**

**Table S1** Baseline patient demographics of the overall HCM cohort stratified by HCM subtype

| **Baseline characteristics** | **Overall HCM  (*n* = 6440)** | **Obstructive HCM**  **(*n* = 3730)** | **Non-obstructive HCM (*n* = 2710)** |
| --- | --- | --- | --- |
| Age, years, mean (SD) | 61.0 (15.6) | 61.2 (15.5) | 60.8 (15.7) |
| Male, *n* (%) | 3988 (61.9) | 2257 (60.5) | 1731 (63.9) |
| White, *n* (%) | 5094 (79.1) | 3013 (80.8) | 2081 (76.8) |
| Asian/British Asian ethnicity, *n* (%) | 583 (9.1) | 333 (8.9) | 250 (9.2) |
| Black/Black British ethnicity, *n* (%) | 467 (7.3) | 233 (6.3) | 234 (8.6) |
| Mixed ethnicity, *n* (%) | 76 (1.2) | 43 (1.2) | 33 (1.2) |
| Other ethnicity, *n* (%) | 109 (1.7) | 60 (1.6) | 49 (1.8) |
| Age-adjusted CCI score, mean (SD) | 2.94 (2.39) | 2.96 (2.40) | 2.90 (2.37) |
| Patients with at least one documented medical condition^a^ | 5036 (78.2) | 2937 (78.7) | 2099 (77.5) |
| Asthma, *n* (%) | 808 (12.6) | 481 (12.9) | 327 (12.1) |
| AF or flutter, *n* (%) | 956 (14.8) | 573 (15.4) | 383 (14.1) |
| Cardiac arrest, *n* (%) | 65 (1.0) | 40 (1.1) | 25 (0.9) |
| Cardiac dysrhythmias^b^,  *n* (%) | 731 (11.4) | 431 (11.6) | 300 (11.1) |
| CKD, *n* (%) | 667 (10.4) | 394 (10.6) | 273 (10.1) |
| Conduction disorders,  *n* (%) | 544 (8.5) | 317 (8.5) | 227 (8.4) |
| COPD, *n* (%) | 465 (7.2) | 287 (7.7) | 178 (6.6) |
| Deep vein thrombosis/pulmonary embolism, *n* (%) | 85 (1.3) | 56 (1.5) | 29 (1.1) |
| Depression, *n* (%) | 521 (8.1) | 327 (8.8) | 194 (7.2) |
| Dilated cardiomyopathy, *n* (%) | 104 (1.6) | 60 (1.6) | 44 (1.6) |
| Heart transplantation, *n* (%) | NR^c^ | NR^c^ | NR^c^ |
| Hypertension, *n* (%) | 3112 (48.3) | 1778 (47.7) | 1334 (49.2) |
| ICD insertion, *n* (%) | 148 (2.3) | 106 (2.8) | 42 (1.6) |
| Ischaemic heart disease, *n* (%) | 1631 (25.3) | 956 (25.6) | 675 (24.9) |
| Myocardial infarction,  *n* (%) | 504 (7.8) | 296 (7.9) | 208 (7.7) |
| Obstructive HCM family history, *n* (%) | 87 (1.4) | 65 (1.7) | 22 (0.8) |
| Pacemaker, *n* (%) | 227 (3.5) | 146 (3.9) | 81 (3.0) |
| Peripheral vascular disease, *n* (%) | 100 (1.6) | 56 (1.5) | 44 (1.6) |
| SRT, *n* (%) | 6 (0.1) | 6 (0.2) | 0 (0.0) |
| Stroke, *n* (%) | 719 (11.2) | 405 (10.9) | 314 (11.6) |
| T1DM, *n* (%) | 50 (0.8) | 29 (0.8) | 21 (0.8) |
| T2DM, *n* (%) | 826 (12.8) | 460 (12.3) | 366 (13.5) |
| Unspecified diabetes,  *n* (%) | 51 (0.8) | 33 (0.9) | 18 (0.7) |
| Transient ischaemic attack, *n* (%) | 107 (1.7) | 58 (1.6) | 49 (1.8) |
| Ventricular assist device, *n* (%) | 0 (0) | 0 (0) | 0 (0) |
| Follow-up, years, mean [SD] | 4.8 (3.0) | 5.2 (3.0) | 4.4 (2.8) |

AF, atrial fibrillation; CCI, Charlson's Comorbidity Index; CKD: chronic kidney disease; COPD, chronic obstructive pulmonary disease; HCM, hypertrophic cardiomyopathy; ICD, implantable cardioverter‑defibrillator; NR, not reported; SD, standard deviation; SRT, septal reduction therapy;
T1DM, type 1 diabetes mellitus; T2DM, type 2 diabetes mellitus.

Ethnicities will not equate to the sum total owing to missing/unknown data.

Unspecified HCM cohort was included within the non-obstructive HCM cohort. The 22 patients who had a diagnosis of non-obstructive HCM followed by a diagnosis of obstructive HCM during the follow-up period were assigned to the obstructive HCM cohort only for the purpose of this analysis.

^a^Documented medical conditions that occurred in the 2 years before index obstructive HCM diagnosis, based on a pre-specified list informed by expert clinical opinion of the most common comorbid conditions observed in clinics considered relevant to understand the clinical and economic burden of HCM. These include asthma, AF or flutter, cardiac arrest, cardiac dysrhythmias, CKD, conduction disorders, COPD, previous deep vein thrombosis/pulmonary embolism, depression, dilated cardiomyopathy, heart transplant, ventricular assist device, previous stroke or transient ischaemic attack, SRT, peripheral vascular disease, ischaemic heart disease, myocardial infarction, T1DM, T2DM, ICD insertion, pacemaker, hypertension, obstructive HCM family history. Patients may have had comorbid medical conditions; therefore, the listed medical conditions do not equate to the sum total of ‘Patients with at least one documented medical condition’.

^b^includes paroxysmal tachycardia (ICD-10: I47), atrial fibrillation and flutter (ICD-10: I48), and other cardiac arrhythmias (ICD-10: I49).

^c^Primary suppression applied to cells with < 5 observations.

Table S2 Baseline active prescriptions for medication in the overall HCM cohort stratified by HCM cohort

| **Medication, *n* (%)** | **Overall HCM**  **(*n* = 6440)** | **Obstructive HCM  (*n* = 3730)** | **Non-obstructive HCM (*n* = 2710)** |
| --- | --- | --- | --- |
| Patients with no known baseline active prescription | 1313 (20.4) | 682 (18.3) | 631 (23.3) |
| Patients with at least one known baseline active prescription | 5127 (79.6) | 3048 (81.7) | 2079 (76.7) |
| Blood-pressure lowering | 4784 (74.3) | 2866 (76.8) | 1918 (70.8) |
| Amiodarone | 283 (4.4) | 193 (5.2) | 90 (3.3) |
| Statins | 3002 (46.6) | 1760 (47.2) | 1242 (45.8) |
| Antiplatelet | 2198 (34.1) | 1291 (34.6) | 907 (33.5) |
| Anticoagulant | 1005 (15.6) | 599 (16.1) | 406 (15.0) |
| Aspirin | 2018 (31.3) | 1194 (32.0) | 824 (30.4) |

HCM, hypertrophic cardiomyopathy.
Unspecified HCM cohort was included within the non-obstructive HCM cohort. The 22 patients who had a diagnosis of non-obstructive HCM followed by a diagnosis of obstructive HCM during the follow-up period were assigned to the obstructive HCM cohort only for the purpose of this analysis.

Baseline active prescriptions were based on prescribing dates before the index date within a 6-week look-back period. The look-back period for all other characteristics and outcomes is 2 years before the index date.

**Table S3** Top 20 initial treatments observed in the obstructive HCM cohort

| **Treatment** | **Patients, *n*** | **Patients, %** |
| --- | --- | --- |
| BB – bisoprolol | 1995 | 64.7 |
| BB – atenolol | 282 | 9.1 |
| CCB – verapamil | 260 | 8.4 |
| CCB – diltiazem | 139 | 4.5 |
| BB – nebivolol | 47 | 1.5 |
| BB – bisoprolol + disopyramide | 46 | 1.5 |
| BB – propranolol | 46 | 1.5 |
| BB – carvedilol | 40 | 1.3 |
| BB – metoprolol | 40 | 1.3 |
| BB – bisoprolol + CCB – diltiazem | 37 | 1.2 |
| BB – bisoprolol + CCB – verapamil | 22 | 0.7 |
| BB – atenolol + BB – bisoprolol | 19 | 0.6 |
| Disopyramide | 18 | 0.6 |
| BB – atenolol + disopyramide | 15 | 0.5 |
| CCB – verapamil + disopyramide | 14 | 0.5 |
| BB – atenolol + CCB – diltiazem | 13 | 0.4 |
| BB – nadolol | 5 | 0.2 |
| CCB – diltiazem + disopyramide | 5 | 0.2 |
| BB – atenolol + CCB – verapamil | NR^a^ | NR^a^ |
| BB – bisoprolol + BB – propranolol | NR^a^ | NR^a^ |

BB, beta-blocker; CCB, calcium channel blocker; HCM, hypertrophic cardiomyopathy; NR, not reported.
^a^Primary suppression applied to cells with < 5 observations.

Table S4 Incidence rates of events per 100 PY in the obstructive HCM cohort stratified by NYHA class

| **Outcome** | **Incidence rate per 100 PY by NYHA class, mean (95% CI)** | | | |
| --- | --- | --- | --- | --- |
|  | **NYHA class I** | **NYHA class II** | **NYHA class III** | **NYHA class IV** |
| All-cause mortality | 1.13 (0.72–1.78) | 1.48 (0.58–3.80) | 2.47 (0.98–6.26) | 1.81 (0.58–5.72) |
| Heart failure | 1.96 (1.38–2.78) | 2.58 (1.25–5.33) | 12.43 (6.12–25.24) | 33.27 (15.39–71.91) |
| SRT | 0.82 (0.45–1.49) | 0.61 (0.17–2.19) | 0.98 (0.28–3.39) | 1.15 (0.26–5.15) |
| Dilated cardiomyopathy | 0.18 (0.06–0.55) | 0.41 (0.04–4.17) | 0.87 (0.09–8.58) | 3.70 (0.36–38.15) |
| Ischaemic stroke | 6.10 (4.55–8.18) | 16.95 (9.39–30.61) | 23.65 (13.07–42.81) | 34.27 (18.34–64.05) |
| AF or flutter | 5.96 (4.80–7.41) | 6.42 (4.08–10.10) | 11.61 (7.41–18.18) | 19.41 (11.3–33.35) |
| Pacemaker | 2.75 (2.02–3.74) | 3.32 (1.75–6.30) | 5.60 (2.98–10.52) | 8.13 (3.87–17.05) |
| ICD insertion | 1.81 (1.25–2.61) | 3.56 (1.67–7.57) | 3.05 (1.43–6.51) | 4.86 (2.00–11.80) |
| Myocardial infarction | 3.00 (2.17–4.14) | 5.19 (2.67–10.09) | 11.91 (6.10–23.22) | 21.59 (9.91–47.00) |
| Deep vein thrombosis or pulmonary embolism | 1.21 (0.69–2.12) | 0.98 (0.31–3.15) | 2.03 (0.62–6.68) | 8.49 (1.21–59.42) |
| Cardiac arrest or ventricular fibrillation | 0.71 (0.39–1.31) | 0.84 (0.24–2.88) | 1.32 (0.37–4.70) | 1.40 (0.30–6.51) |
| Ventricular tachycardia | 0.35 (0.15–0.81) | 2.79 (0.53–14.81) | 2.88 (0.54–15.33) | 5.78 (0.99–33.76) |
| Conduction disorders | 2.81 (2.08–3.80) | 3.55 (1.89–6.65) | 6.32 (3.40–11.75) | 9.60 (4.67–19.73) |
| Cardiac dysrhythmias | 3.77 (2.90–4.90) | 7.05 (4.10–12.12) | 10.23 (5.98–17.52) | 18.91 (10.32–34.65) |

AF, atrial fibrillation; CI, confidence interval; HCM, hypertrophic cardiomyopathy;
ICD, implantable cardioverter‑defibrillator; NYHA, New York Heart Association; PY, patient-years;
SRT, septal reduction therapy.

Incidence rates were not calculated for heart transplant owing to low total number of events.

Table S5 Healthcare resource use for patients with obstructive HCM, stratified by NYHA class

| **Consultation type** | **Unit cost, £** | **NYHA class I**  **(*n* = 1039/PY = 1709)** | | | | **NYHA class II**  **(*n* = 2585/PY = 8565)** | | | | **NYHA class III**  **(*n* = 2418/PY = 8296)** | | | | **NYHA class IV**  **(*n* = 404/PY = 782)** | | | |
| --- | --- | --- | --- | --- | --- | --- | --- | --- | --- | --- | --- | --- | --- | --- | --- | --- | --- |
|  |  | ***n*** | ***n* per**  **1000 PY** | **Total cost, £** | **Cost per PY, £** | ***n*** | ***n* per**  **1000 PY** | **Total cost, £** | **Cost per PY, £** | ***n*** | ***n* per**  **1000 PY** | **Total cost, £** | **Cost per PY, £** | ***n*** | ***n* per**  **1000 PY** | **Total cost, £** | **Cost per PY, £** |
| **Primary care**^a^ | | | | | | | | | | | | | | | | | |
| All | 30.78^b^ | 11,628^c^ | 6803.98 | 357,909.84 | 209.43 | 87,142 | 10,174.20 | 2,682,230.76 | 313.16 | 124,580 | 15,016.88 | 3,834,572.40 | 462.22 | 16,496 | 21,094.63 | 507,746.88 | 649.29 |
| General practice | 39.23 | 6458 | 3778.82 | 253,347.34 | 148.24 | 47,130 | 5502.63 | 1,848,909.90 | 215.87 | 65,909 | 7944.67 | 2,585,610.07 | 311.67 | 8317 | 10,635.55 | 326,275.91 | 417.23 |
| Nurse practice | 14.06 | 3230 | 1889.99 | 45,412.27 | 26.57 | 25,072 | 2927.26 | 352,500.43 | 41.16 | 37,306 | 4496.87 | 524,504.67 | 63.22 | 5265 | 6732.74 | 74,023.40 | 94.66 |
| Telephone | 28.21 | 1940 | 1135.17 | 54,722.81 | 32.02 | 14,940 | 1744.31 | 421,422.07 | 49.20 | 21,365 | 2575.34 | 602,656.13 | 72.64 | 2914 | 3726.34 | 82,197.05 | 105.11 |
| Out-of-hours practice (not included elsewhere) | 136.77 | 47 | 27.50 | 6428.19 | 3.76 | 355 | 41.45 | 48,553.35 | 5.67 | 472 | 56.89 | 64,555.44 | 7.78 | 49 | 62.66 | 6701.73 | 8.57 |
| **Secondary care^d^** | | | | | | | | | | | | | | | | | |
| Inpatient (all)^e^ | - | - | - | 2,770,419.65 | 1621.08 | - | - | 13,595,573.52 | 1587.34 | - | - | 20,605,153.74 | 2483.75 | - | - | 3,112,145.43 | 3979.73 |
| Day case visits | - | - | - | 499,471.58 | 292.26 | - | - | 3,019,670.44 | 352.56 | - | - | 3,826,409.25 | 461.24 | - | - | 408,364.46 | 522.21 |
| Inpatient (elective – excluding day cases) | - | - | - | 1,271,352.32 | 743.92 | - | - | 4,393,067.85 | 512.91 | - | - | 6,068,558.32 | 731.50 | - | - | 704,344.74 | 900.70 |
| Inpatient (non-elective – excluding day cases) | - | - | - | 999,595.75 | 584.90 | - | - | 6,182,835.23 | 721.87 | - | - | 10,710,186.17 | 1291.01 | - | - | 1,999,436.23 | 2556.82 |
| Outpatient visits | 137 | 7683 | 4495.61 | 1,052,571.00 | 615.90 | 52,809 | 6165.67 | 7,234,833.00 | 844.70 | 66,659 | 8035.08 | 9,132,283.00 | 1100.81 | 8597 | 10,993.61 | 1,177,789.00 | 1506.12 |
| A&E visits | 188.28 | 712 | 416.62 | 134,055.36 | 78.44 | 4496 | 524.93 | 846,506.88 | 98.83 | 6248 | 753.13 | 1,176,373.44 | 141.80 | 928 | 1186.70 | 174,723.84 | 223.43 |
| Inpatient critical care | 1215.90 | 56 | 32.77 | 68,090.40 | 39.84 | 240 | 28.02 | 291,816.00 | 34.07 | 438 | 52.80 | 532,564.20 | 64.20 | 56 | 71.61 | 68,090.40 | 87.07 |
| **Test and procedures^f^** | | | | | | | | | | | | | | | | | |
| Ablation | 6750.00 | 17 | 9.95 | 114,750.00 | 67.14 | 139 | 16.23 | 938,250.00 | 109.54 | 139 | 16.76 | 938,250.00 | 113.10 | 27 | 34.53 | 182,250.00 | 233.06 |
| Biomarker: NT-proBNP | 20.00 | 15 | 8.78 | 300.00 | 0.18 | 192 | 22.42 | 3840.00 | 0.45 | 477 | 57.50 | 9540.00 | 1.15 | 86 | 109.97 | 1720.00 | 2.20 |
| Biomarker: Troponin T | 20.00 | NR ^g^ | NR^h^ | NR^h^ | NR^h^ | 41 | 4.79 | 820.00 | 0.10 | 71 | 8.56 | 1420.00 | 0.17 | 12 | 15.35 | 240.00 | 0.31 |
| Cardiac MRI | 451.49 | 80 | 46.81 | 36,119.20 | 21.13 | 84 | 9.81 | 37,925.16 | 4.43 | 104 | 12.54 | 46,954.96 | 5.66 | 5 | 6.39 | 2257.45 | 2.89 |
| Cardiac rehabilitation | 374.54 | 7 | 4.10 | 2621.78 | 1.53 | 18 | 2.10 | 6741.72 | 0.79 | 32 | 3.86 | 11,985.28 | 1.44 | 7 | 8.95 | 2621.78 | 3.35 |
| ECG | 130.26 | 55 | 32.18 | 7164.30 | 4.19 | 241 | 28.14 | 31,392.66 | 3.67 | 238 | 28.69 | 31,001.88 | 3.74 | 25 | 31.97 | 3256.50 | 4.16 |
| ECHO | 191.27 | 172 | 100.64 | 32,898.44 | 19.25 | 821 | 95.86 | 157,032.67 | 18.33 | 1401 | 168.88 | 267,969.27 | 32.30 | 218 | 278.77 | 41,696.86 | 53.32 |
| ECHO stress induced | 130.26 | 0 | 0.00 | 0.00 | 0.00 | NR^g^ | NR^h^ | NR^h^ | NR^h^ | 0 | 0.00 | 0.00 | 0.00 | 0 | 0.00 | 0.00 | 0.00 |
| ICD insertion and cardiac resynchronization therapy | 3191.62 | 47 | 27.50 | 150,006.14 | 87.77 | 656 | 76.59 | 2,093,702.72 | 244.45 | 601 | 72.20 | 1,918,163.62 | 230.45 | 90 | 112.53 | 287,245.80 | 367.32 |
| Pacemaker | 3068.66 | 96 | 56.17 | 294,591.36 | 172.38 | 684 | 79.86 | 2,098,963.44 | 245.06 | 1129 | 136.09 | 3,464,517.14 | 417.61 | 160 | 204.60 | 490,985.60 | 627.86 |
| SRT-myectomy | 15,674.75 | 6 | 3.51 | 94,048.50 | 55.03 | 22 | 2.57 | 344,844.50 | 40.26 | 36 | 4.34 | 564,291.00 | 68.02 | NR^g^ | NR^h^ | NR^h^ | NR^h^ |
| SRT-ASA | 6750.00 | 10 | 5.85 | 67,500.00 | 39.50 | 31 | 3.62 | 209,250.00 | 24.43 | 63 | 7.59 | 425,250.00 | 51.26 | 7 | 8.95 | 47,250.00 | 60.42 |

A&E, accident and emergency; ECG, electrocardiogram; ECHO, echocardiogram; ICD, implantable cardioverter‑defibrillator; GP, general practitioner; HCM, hypertrophic cardiomyopathy; HES, Hospital Episode Statistics; HRG, Healthcare Resource Group; MRI, magnetic resonance imaging; NHS, National Health Service; NR, not reported; NT-proBNP, N-terminal pro B-type natriuretic peptide; NYHA, New York Heart Association; PSSRU, Personal Social Services Research Unit; PY, patient-years; SRT, septal reduction therapy; SRT-ASA, alcohol septal ablation; SRT-myectomy, myectomy of left ventricular outflow tract.

^a^GP consultation cost as referenced in PSSRU-2021. GP consultation 2021–2016 costs ratio was used to estimate 2021 unit cost for all other PC activities from 2016 values.

^b^Unit cost of all clinical consultations calculated as a weighted average of general practice, nurse practice, and GP telephone consultation costs.

^c^All clinical consultations calculated as a sum of general practice, nurse practice, and GP telephone consultation counts (*n*).

^d^Secondary care unit costs and count were not used to calculate total inpatient costs. Inpatient costs for elective and non-elective inpatient admissions, and day cases were calculated using the HES HRG table to pull the total cost of the relevant inpatient visits using HRG currency codes.

^e^Unit cost of inpatient (all) admissions calculated as a weighted average of inpatient admission (elective – excluding day cases), inpatient (non-elective – excluding day cases), and day case visits.

^f^Unit costs for tests and procedures as provided in the National Schedule of NHS costs – Year 2019-20 – NHS Trust and NHS Foundation Trusts (cost modified based on expert elicitation).

^g^Primary suppression applied to cells with < 5 observations.

^h^Secondary suppression.

Supplementary figures

**Figure S1** Summary of patient counts through each attrition criterion and HCM cohort.

^a^Including hypertensive heart disease, aortic stenosis, athlete’s heart, storage disease, Takotsubo cardiomyopathy, Fabry disease, Anderson–Fabry disease, Pompe’s disease, Amyloidosis.

^b^The 22 patients who had a diagnosis of non-obstructive HCM followed by a diagnosis of obstructive HCM during the follow-up period were assigned to the obstructive HCM cohort only for the purpose of this analysis.

^c^Unspecified HCM cohort was included within the non-obstructive HCM cohort.

CPRD, Clinical Practice Research Datalink; HCM, hypertrophic cardiomyopathy; HES, Hospital Episode Statistics.


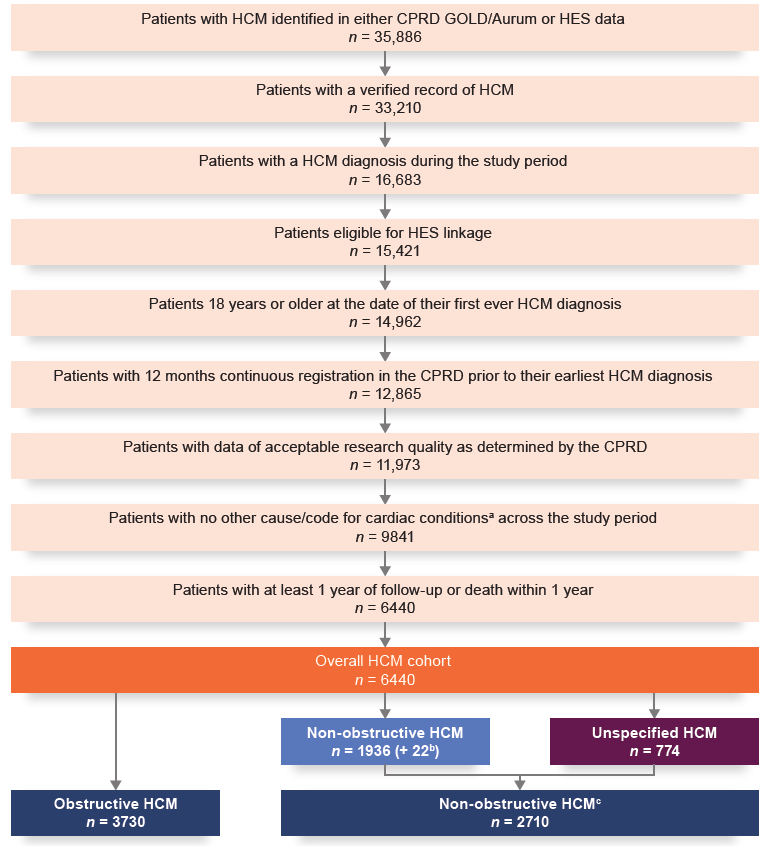


**Figure S2** Cumulative risk of treatment augmentation (A), treatment discontinuation (B), and no treatment (C) in the obstructive HCM cohort by initial treatment observed.

^a^Includes disopyramide as monotherapy and in combination therapy.

BB, beta-blocker; CCB, calcium channel blocker; HCM, hypertrophic cardiomyopathy.


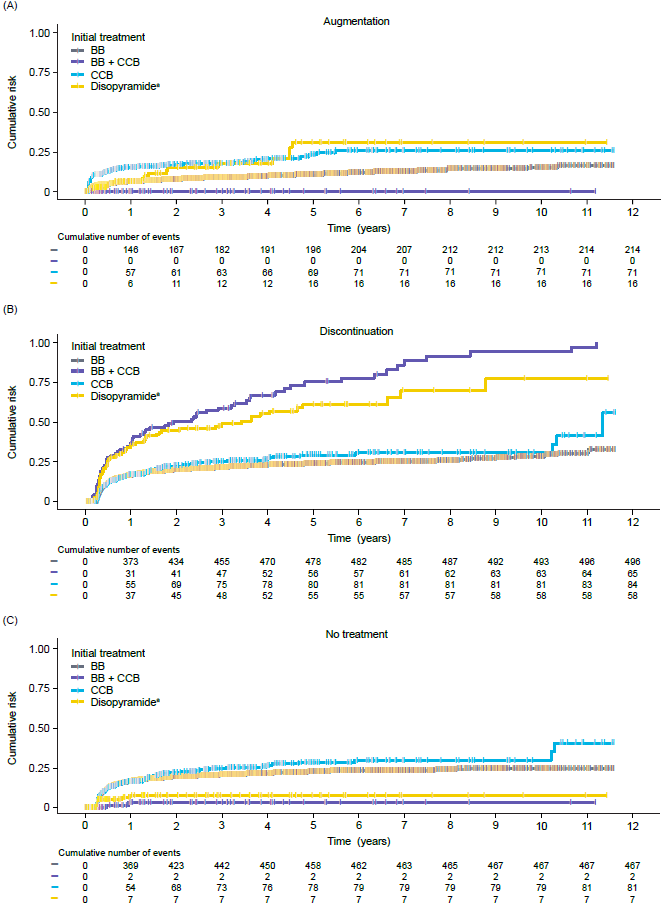

Supplement: Supplementary file 1 — Table S1. Baseline patient demographics of the overall HCM cohort stratified by HCM subtype Table S2. Baseline active prescriptions for medication in the overall HCM cohort stratified by HCM cohort Table S3. Top 20 initial treatments observed in the obstructive HCM cohort Table S4. Incidence rates of events per 100 PY in the obstructive HCM cohort stratified by NYHA class Table S5. Healthcare resource use for patients with obstructive HCM, stratified by NYHA class Figure S1. Summary of patient counts through each attrition criterion and HCM cohort. Figure S2. Cumulative risk of treatment augmentation (A), treatment discontinuation (B), and no treatment (C) in the obstructive HCM cohort by initial treatment observed. [file EHF2-12-2034-s001.docx]
